# Targeting Tim-3 in Cancer With Resistance to PD-1/PD-L1 Blockade

**DOI:** 10.3389/fonc.2021.731175

**Published:** 2021-09-22

**Authors:** Tian Tian, Zhaoming Li

**Affiliations:** ^1^Department of Neurology, The First Affiliated Hospital of Zhengzhou University, Zhengzhou, China; ^2^Department of Oncology, The First Affiliated Hospital of Zhengzhou University, Zhengzhou, China

**Keywords:** Tim-3, PD-1, PD-L1, immune checkpoint, drug resistance

## Abstract

Programmed death receptor 1 (PD-1) or programmed death ligand 1 (PD-L1) blocking therapy has completely changed the treatment pattern of malignant tumors. It has been tested in a wide range of malignant tumors and achieved clinical success. It might be a promising cancer treatment strategy. However, one of the important disadvantages of PD-1/PD-L1 blocking therapy is that only a few patients have a positive response to it. In addition, primary or acquired drug resistance can also lead to cancer recurrence in patients with clinical response. Therefore, it is very important to overcome the resistance of PD-1/PD-L1 blocking therapy and improve the overall response rate of patients to the immunotherapy. T cell immunoglobulin and mucin domain molecule 3 (Tim-3) belongs to the co-inhibitory receptor family involved in immune checkpoint function. Due to adaptive resistance, the expression of Tim-3 is up-regulated in PD-1/PD-L1 blocking therapy resistant tumors. Therefore, blocking the immune checkpoint Tim-3 might antagonize the resistance of PD-1/PD-L1 blocking therapy. This review systematically introduces the preclinical and clinical data of combined blockade of Tim-3 and PD-1/PD-L1 in cancer immunotherapy, and discusses the prospect of overcoming the drug resistance of PD-1/PD-L1 blockade therapy through blockade of Tim-3.

## Introduction

Co-inhibitory receptors play the following important roles in cells: regulating T cell response and maintaining immune homeostasis (1). However, co-inhibitory receptors also limit the ability of T cells to respond effectively to tumors or pathogens. T cells express a variety of co-inhibitory receptors: CTLA-4 (cytotoxic T lymphocyte associated protein 4 or CD152), PD-1 (programmed death ligand 1 or CD279), Tim-3 (T cell immunoglobulin and mucin containing protein 3 or CD366), TIGIT (T cell immune receptor with immunoglobulin and ITIM domains), LAG-3 (lymphocyte activation gene 3 or CD223) and Vista (T cell activation inhibitor containing V domain immunoglobulin) (2–4).

Tim-3 is a type I transmembrane protein which is encoded by gene havcr2 (hepatitis A virus cellular receptor 2) (5, 6). Its extracellular domain is composed of the N-terminal immunoglobulin (IgV) domain at the distal end of the membrane, followed by the membrane mucin domain containing O-linked glycosylation potential (7). Tim-3 is expressed in a variety of immune related cells, such as CD4^+^ and CD8^+^ T cells (5), regulatory T cells (Tregs), natural killer (NK) cells, macrophages, mast cells and dendritic cells (DC) (8–12).

## Ligands and Signaling Pathways of Tim-3

So far, four ligands have been identified to interact with different regions of Tim-3 extracellular immunoglobulin V domain: galectin-9 (Gal-9), phosphatidylserine (PtdSer), high-mobility group protein B1 (HMGB1), and cell adhesion molecule bound to carcinoembryonic antigen 1 (CEACAM1) (2, 13).

**Figure 1:**
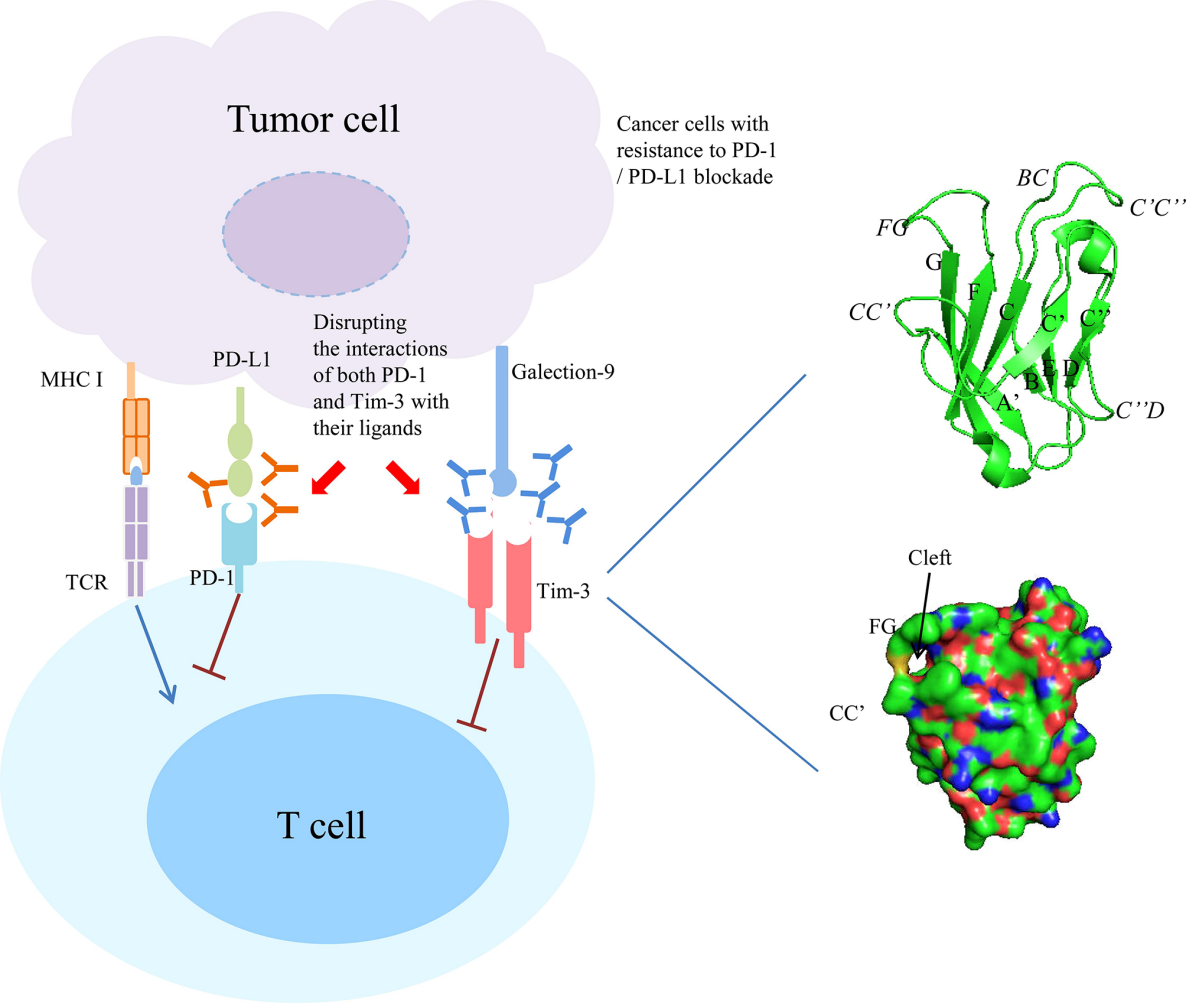
Combined targeting Tim-3 and PD-1 pathway in cancer with resistance to PD-1/PD-L1 blockade. Right panel showed the ribbon diagram and surface view of the of human Tim-3 IgV domain crystal structure. The β strands are labeled with uppercase letters and loops are highlighted in italics.

The Tim-3 IgV domain are composed of two anti-parallel β-sheets with A, G, F, C, C’ and C’ β-strands in one sheet (GFC β-sheet) and the short β-strands, B, E and D in the other sheet (BED β-sheet) (**Figure 1**, right upper panel). It contains six conserved Cys residues, and the first and last of these six Cys residues bridge the β-sheets. The four additional Cys residues form two additional disulfide bonds that fix the long CC’ loop folded upwards onto the GFC β-sheet. The critical feature of Tim-3 IgV domain is a deep binding pocket flanked by two hydrophobic loops that can extend into a membrane (**Figure 1**, right lower panel). The tip of the CC’ loop projects parallel to the FG loop in the IgV domain, generating a pocket that is used for recognition of ligands (6, 14). The binding of Tim-3 to Gal-9 induces the phosphorylation of two key tyrosine residues,Y265 and Y272 (Y256 and Y263 in mice), which in turn promotes the release of BAT3 from the cytoplasmic tail of Tim-3 (15, 16). After BAT3 release, Src kinase binds and promotes the subsequent negative regulation of T-cell receptor (TCR) signal transduction (16, 17). CEACAM1 and Gal-9 bind to different IgV domains, but both ligands induce the phosphorylation of the same two tyrosine residues which are required for the functional activity of Tim-3 (17–25). PtdSer is a non-protein ligand that is shared among different Tim family members and released from apoptotic cells (13, 26–28). It have been reported that Tim-3 recognizes apoptotic cells through the FG loop in the IgV domain (**Figure 1**) (14, 29–34). The last one ligand is HMGB1 (35, 36). Binding of Tim-3 with HMGB1 interfered with the recruitment of nucleic acids into DC endosomes, which lead to the attenuated therapeutic efficacy of DNA vaccination and chemotherapy by diminishing the immunogenicity of nucleic acids released from dying cancer cells (35).

## Tim-3 Expression and Its Role in Regulating Anti-Tumor Immunity

Tim-3 was initially identified as expressed on cytotoxic T cells (Tc1) and T helper type 1 (Th1) cells and acts mainly as a negative regulator of type 1 immunity (5). Tim-3 is also highly expressed in NK cells, macrophages and dendritic cells (37–42). The binding of Gal-9 on Tim-3 promotes the production of IFN-γ by NK cells, while blocking Tim-3 by specific antibody will inhibit IFN-γ production (40). Tim-3 expression on macrophages is down regulated in response to TLR4 stimulation and has an inhibitory effect during sepsis (43). In dendritic cells, the binding of Tim-3 to HMGB1 inhibits dendritic cells activation by interfering with the nucleic acid sensing (35).

Increased expression of Tim-3 in human tumors, particularly on immune cells, might be a potential prognostic biomarker for a variety of tumors. For example, in patients with hepatitis B virus related hepatocellular carcinoma (HCC), the expression of Tim-3 on CD4^+^ and CD8^+^ T cells was increased. Tim-3^+^ T cells were replicative senescent and expressed surface and genetic markers for senescence (44–46). In addition, the number of tumor infiltrating cells in Tim-3^+^ was negatively correlated with the survival rate of HCC patients (47, 48). In prostate cancer, the expression of Tim-3 is higher than that of adjacent benign tissues, and the high expression of Tim-3 is an independent predictor of recurrence free and progression free survival (49–53). It has been shown that the abnormal expression of Tim-3 in tumor was closely related to the depletion of T cells (52, 54–57). For example, in a variety of mouse tumor models, Tim-3 is widely expressed on CD8^+^ tumor infiltrating lymphocytes (58). In mice bearing the solid tumor CT26 colon carcinoma, it shows that among CD8+ TILs, cells that coexpress Tim-3 and PD-1 comprise the major population (∼50%) with cells expressing PD-1 alone or neither Tim-3 nor PD-1 comprising smaller populations (∼30% and ∼20%, respectively) (59).This phenomena was also observed in mice bearing two other solid tumors: 4T1 mammary adenocarcinoma and B16F10 melanoma (59). Tim-3^+^ PD-1+ tumor infiltrating lymphocytes showed the most severe failure phenotype, which was characterized by the inability to proliferate and produce IFN-γ, IL-2 and TNF-α (44, 51, 58–61).

Tim-3 also participates in the progression of tumor by regulating Tim-3^+^ Foxp3^+^ Treg cells and innate immune cells (62–65). For example, Tim-3^+^ Foxp3^+^ CD4^+^ cells are widely found in non-small-cell lung carcinoma (NSCLC), HCC, cervical, colorectal and ovarian cancer et al. (66–70). In NSCLC, about 70% of Tim-3^+^ CD4^+^TILs expressed Foxp3, and about 60% of Foxp3^+^ tumor infiltrating lymphocytes were Tim-3 positive (9, 71). It is important that the T cells expressed Tim-3 on CD4^+^ are associated with lymph node metastasis and advanced cancer staging (62). In patients with HCC, the expression of Tim-3 in peripheral blood mononuclear cells and tumor-associated macrophages (TAM) increased significantly, and that is closely related to higher tumor grade and poor survival for patients with HCC (44, 72, 73). In addition, the interference of Tim-3 in macrophages significantly inhibited the alternative activation of macrophages, and inhibited the growth of HCC cells *in vitro* and *in vivo* (44, 72, 73).

In addition, Tim-3 was also directly expressed in tumor cells (62). For example, Tim-3 is expressed in osteosarcoma and triggers tumor cells to obtain aggressive EMT characteristics (62). In clear cell renal cell carcinoma, Tim-3 was expressed on cancer cells and CD204^+^ tumor related macrophages. The higher expression level of Tim-3 was positively correlated with the short progression free survival (PFS) of patients with clear cell renal cell carcinoma (74). In acute myeloid leukemia (AML), Tim-3 was not expressed on normal hematopoietic stem cells, but mainly on leukemic stem cells in most types of AML (75). Recently, a number of studies have found that the mutation of Tim-3 might be related to the occurrence of subcutaneous panniculitis like T-cell lymphoma (76–79).

## Rationale for Targeting Both PD-1 and Tim-3

Cancer immunotherapy with monoclonal antibodies to PD-1 and PD-L1 has achieved significant therapeutic effects in various cancers (80–84). However, it is worth noting that patients who receive anti PD-1 or anti PD-L1 monoclonal antibody treatment will confront with the drug resistance problems, which leads to cancer recurrence in many patients (85).

First, in the chronic lymphocytic choriomeningitis virus infection, virus-specific CD8 T cells retained high Tim-3 expression throughout chronic infection. The majority (65% to 80%) of lymphocytic choriomeningitis virus-specific CD8 T cells in lymphoid and nonlymphoid organs coexpressed Tim-3 and PD-1. This coexpression was associated with more severe CD8 T-cell exhaustion in terms of proliferation and secretion of effector cytokines such as IFN-gamma, TNF-alpha, and IL-2. Interestingly, CD8 T cells expressing both inhibitory receptors also produced the suppressive cytokine IL-10. Most importantly, combined blockade of Tim-3 and PD-1 pathways *in vivo* synergistically improved CD8 T cell responses and viral control in chronically infected mice. Taken together, it suggests that targeting both PD-1 and Tim-3 is an effective immune strategy for treating chronic viral infections.

Second, Anderson et al. found that CD8+ TILs that coexpress Tim-3 and PD-1 not only represent the most abundant TIL population in multiple solid tumors but also represent the most dysfunctional or exhausted population of TILs (59). They treated CT26 tumor-bearing mice with an anti–Tim-3 antibody, anti-PD-L1 antibody, anti–Tim-3 plus anti–PD-L1 antibodies, or control immunoglobulins (59). They found that treatment with anti–Tim-3 alone had little or no effect and treatment with anti–PD-L1 alone showed a trend toward delayed tumor growth, but this varied between experiments and did not reach statistical significance. However, combined treatment with anti–Tim-3 and anti–PD-L1 resulted in a dramatic reduction in tumor growth, with 50% of the mice exhibiting complete tumor regression (59). Similarly, a recent study has found that simultaneous targeting of the Tim-3 and PD-1 pathways also rescues CD8+ T cells from exhaustion in a model of chronic infection (85). Together, these findings support combined targeting of the Tim-3 and PD-1 pathways as an effective treatment not only for cancer but also for other chronic immune conditions where T cell exhaustion is known to occur.

Third, a preclinical investigation evaluated the effects of dual PD-1 and Tim-3 blockade with radiation in human glioblastoma multiforme. C57BL/6 mice were implanted with murine glioma cell line GL261-luc2 and randomized into 8 treatment arms: (i) control, (ii) SRS(stereotactic radiosurgery), (iii) anti-PD-1 antibody, (iv) Tim-3 antibody, (v) anti-PD-1 + SRS, (vi) Tim-3 + SRS, (vii) anti-PD-1 + Tim-3, and (viii) anti-PD-1 + Tim-3 + SRS. It showed that neither Tim-3 nor SRS alone had significant treatment effect, whereas anti-PD-1 improved median survival (33 days) compared with control (22 days, P < 0.0001). Adding Tim-3 to anti-PD-1 therapy improved median survival from 33 days (anti-PD-1 alone) to 100 days (Tim-3 + anti-PD-1) and improved overall survival (OS) from 27.8% to 57.9%, respectively (86). Therefore, combined targeting of the Tim-3 and PD-1 pathways is more effective in suppressing tumor growth than any single target pathway.

Last, another independent study used two genetically engineered mouse models of lung adenocarcinomas corresponding to the two most common oncogene drivers in human lung adenocarcinoma, KRAS and EGFR. The EGFR and Kras models were treated with a therapeutic anti-PD-1 antibody until tumors demonstrated progression by magnetic resonance imaging and evaluated immune profiles. It found that upregulation of other immune checkpoints, most notably Tim-3, on therapeutic antibody-bound T cells as a marker of treatment resistance. To determine whether blockade of Tim-3 at the time of resistance might be therapeutically efficacious, TIM-3-blocking treatment in these mice were performed and demonstrated a clinical benefit. Moreover, to extend these results and determine their applicability to patients treated with anti-PD-1 antibodies, specimens from two patients who showed an initial response to PD-1 blockade but ultimately developed progressive disease were analyzed. These cases exhibited similar upregulation of Tim-3 on therapeutic antibody-bound TILs. These results suggest that targeting alternate immune checkpoints such as Tim-3 upregulated in the context of PD-1 therapy may extend the benefit of PD-1 blockade in responsive tumors (87) (**Figure 1**).

## Clinical Trials of Targeting Both PD-1 and Tim-3 in Human Tumors

At present, there are several ongoing clinical trials to explore the application of combined blocking Tim-3 and PD-1 in advanced solid tumors (**Table 1**).

**Table 1:** Main ongoing clinical trials of anti-Tim-3 combined with anti-PD-1 antibody.

NCT Number	Title	Tumor type	Interventions	Phases
NCT03680508	TSR-022 and TSR-042 in Patients With Liver Cancer	Liver Cancer	Drug: TSR-022 and TSR-042	Phase II
NCT04139902	Neoadjuvant PD-1 Inhibitor Dostarlimab (TSR-042) vs. Combination of TIM-3 Inhibitor TSR-022 and PD-1 Inhibitor Dostarlimab (TSR-042) in Melanoma	Melanoma	Drug: Dostarlimab (TSR-042) (singly)|Drug: Dostarlimab (TSR-042) and TSR-022 (combination)	Phase II
NCT03708328	A Dose Escalation and Expansion Study of RO7121661, a PD-1/TIM-3 Bispecific antibody, in Participants With Advanced and/or Metastatic Solid Tumors	Solid Tumors	Drug: RO7121661	Phase I
NCT04931654	A Study to Assess the Safety and Efficacy of AZD7789 in Participants With Advanced or Metastatic Solid Cancer	Non-Small-Cell Lung cancer	Drug: AZD7789	Phase I/II
NCT04370704	Study of Combination Therapy With INCMGA00012, INCAGN02385, and INCAGN02390 in Participants With Select Advanced Malignancies	Melanoma	Drug: INCAGN02385|Drug: INCAGN02390|Drug: INCMGA00012.	Phase I/II
NCT03961971	Trial of Tim-3 in Combination With anti-PD-1 and SRS in Recurrent GBM	Glioblastoma Multiforme	Drug: sabatomimab	Phase I
NCT03311412	A Phase 1, Open-Label, Multicenter Trial Investigating the Safety, Tolerability, and Preliminary antineoplastic Activity of Sym021 as Monotherapy, in Combination With Either Sym022 or Sym023, and in Combination With Both Sym022 and Sym023 in Patients With Advanced Solid Tumor Malignancies or Lymphomas	Metastatic Cancer, Solid Tumor, Lymphoma	Drug: Sym021|Drug: Sym022|Drug: Sym023	Phase I
NCT03744468	Study of BGB-A425 in Combination With Tislelizumab in Advanced Solid Tumors	Solid Tumors	Drug: BGB-A425|Drug: tislelizumab	Phase I/II
NCT04641871	Sym021 in Combination With Either Sym022 or Sym023 in Patients With Advanced Solid Tumor Malignancies	Solid Tumors	Drug: Sym021|Drug: Sym022|Drug: Sym023	Phase I
NCT04785820	A Study of RO7121661 and RO7247669 Compared With Nivolumab in Participants With Advanced or Metastatic Squamous Cell Carcinoma of the Esophagus	Esophageal Squamous Cell Carcinoma	Drug: RO7121661|Drug: RO7247669|Drug: Nivolumab	Phase II
NCT02817633	A Study of TSR-022 in Participants With Advanced Solid Tumors (AMBER)	Solid Tumors	Drug: TSR-022,Nivolumab	Phase I
NCT02608268	Phase I-Ib/II Study of sabatomimab as Single Agent and in Combination With PDR001 in Patients With Advanced Malignancies	Advanced Malignancies	Drug: Sabatomimab|Drug: PDR001|Drug: Decitabine	Phase I/II

TSR-022, Tim-3 antibody; TSR-042, anti-PD-1 antibody; RO7121661, PD1-Tim-3 bispecific antibody; RO7247669, PD1-LAG3 bispecific antibody; AZD7789, anti-PD-1 and Tim-3 bispecific antibody; INCMGA00012, anti-PD-1 antibody; INCAGN02385, anti-LAG-3 antibody; INCAGN02390, Tim-3 antibody; BGB-A425, humanized immunoglobulin gamma-1 (IgG1)-variant monoclonal antibody against Tim-3; Tislelizumab, humanized immunoglobulin G4 (IgG4)-variant monoclonal antibody against PD-1; TSR-022,Tim-3 antibody; Sabatomimab, Tim-3 antibody.

A phase I/II study evaluated the safety and efficacy of sabatomimab with or without spartalizumab in patients with advanced solid tumors (88). Among 219 patients, the most common were ovarian cancer (17%) and colorectal cancer (7%). 133 patients were treated with sabatolimab, 86 patients were treated with sabatomimab combined with spartalizumab. The most common adverse event suspected to be treatment-related was fatigue (9%, sabatolimab; 15%, combination). No responses were seen with sabatolimab. Five patients receiving combination treatment had partial responses (6%; lasting 12-27 months) in colorectal cancer (n = 2), non-small cell lung cancer (NSCLC), malignant perianal melanoma, and SCLC. Of the five, two patients had elevated expression of immune markers in baseline biopsies; another three had >10% TIM-3-positive staining, including one patient with NSCLC who received prior PD-1 therapy (88). It suggested that sabatomimab combined with spartalizumab was well tolerated and showed preliminary signs of antitumor activity.

LY3321367 is a novel Tim-3 monoclonal antibody. An open label, multicenter, phase Ia/b solid tumor study explored the safety, tolerability, recommended phase II dose, pharmacokinetics/pharmacodynamics, immunogenicity and efficacy of LY3321367 alone or in combination with anti-PD-L1 antibody LY300054 (89). No dose limiting toxicity was observed in the dose escalation of monotherapy (n = 30) or combination therapy (n = 28). The treatment-related adverse events of LY3321367 (≥2 patients) mainly included pruritus, fatigue, rash, anorexia and infusion related reactions. In the non-small cell lung cancer monotherapy expansion cohort, outcomes varied by prior anti-PD-1 therapy response status: anti-PD-1/L1 refractory patients (m= 23, objective response rate (ORR) 0%, disease control rate (DCR) 35%, progression-free survival (PFS) 1.9 months) versus anti-PD-1/L1 responders (n = 14, ORR 7%, DCR 50%, PFS 7.3 months). In combination expansion cohorts (n = 91), ORR and DCR were 4% and 42% (89). LY3321367 showed acceptable safety and good pharmacokinetics/pharmacodynamics, but its anti-tumor activity needs further study. Besides, this study has limitations. The small sample size and the unselected enrollment of patients might result in statistical bias and limit the statistical power of this study to some degree.

LY3415244 is a bispecific antibody against Tim-3/PD-L1. A phase I, multicenter, open label study evaluated the safety and efficacy of combined blockade of Tim-3 and PD-L1 in patients with advanced solid tumors (90). A total of 12 patients were included in this study and received at least one dose of LY3415244. Two patients (16.7%) developed clinically significant anaphylactic infusion-related reactions and all patients developed treatment-emergent antidrug antibodies (TE-ADA). ADA titers were sometimes very high and negatively impacted soluble TIM-3 target engagement in most patients. ADA epitope specificity was against both TIM-3 and PD-L1 arms of the bispecific antibody (90). This TIM-3 and PD-L1 bispecific format was associated with unexpected immunogenicity targeting both arms of the bispecific antibody, which might be responsible for the early study termination. Therefore, this experience emphasizes the importance of thorough analyses for preexisting ADAs as part of immunogenicity risk assessment of novel antibodies.

In addition, sabatomimab and spartalizumab are humanized IgG4 monoclonal antibodies. Sabatomimab blocks the binding of Tim-3 to PtdSer, while spartalizumab blocks the binding of PD-1 to PD-L1/2. A phase II clinical trial (NCT02608268) explored the dose expansion of sabatomimab combined with spartalizumab in patients with NSCLC and melanoma. Thirty three patients received combination therapy, including 16 patients with melanoma and 17 patients with NSCLC. The preliminary results showed that sabatomimab combined with spartalizumab was well tolerated, but the efficacy of combination of sabatomimab and spartalizumab in patients with melanoma and NSCLC needs further data (91).

## Conclusion and Perspectives

The activation of naive T cells requires both the stimulation of the TCR by MHC-peptide complex and co-stimulatory signaling by co-stimulatory receptors with their corresponding ligands on antigen-presenting cells (92–95). There are stimulatory and inhibitory co-receptors on the cell-surface which positively or negatively regulate TCR driven signals, respectively (95). To date, many co-stimulatory receptors have been identified including CD28, ICOS, 4-1BB, CD226, OX-40, and GITR (95). For example, the co-stimulatory receptor CD28 on T cells and its ligand B7-1 or B7-2 on activated APCs amplify TCR signaling, leading to T-cell proliferation and IL-2 production (95, 96). When T cells are being activated and expanded, the expression of co-inhibitory receptors is up-regulated. Co-inhibitory receptors includes PD-1, CTLA-4, TIM-3, LAG-3, and TIGIT (95). They play an important role in activated T cells, regulatory T cells, and exhausted T cells. These receptors suppress T-cell function in the tumor microenvironment, thereby making the T cells dysfunctional. Therefore, blockade of co-inhibitory receptors (such as PD-1) has emerged as a successful treatment option for a number of human cancers (97). However, primary or acquired drug resistance may eventually lead to cancer progression in patients with clinical response (3). Therefore, resistance to PD-1/PD-L1 blockade remains a major challenge to its further application. Accumulating evidence supports the importance of targeting Tim-3 in the treatment of cancer. Importantly, a number of preclinical studies have shown that, compared with the use of anti-PD-1 antibody alone, the combined treatment of blocking Tim-3 and PD-1 can significantly improve the survival rate of mice (59, 87). Moreover, several ongoing clinical trials have also evaluated the safety and efficacy of the combination therapy (88, 89). Therefore, the combined blockade of Tim-3 and PD-1 pathway might be a promising strategy for tumor immunotherapy.

Although a number of preclinical and clinical studies have shown that, compared with the use of anti-PD-1 antibody alone, the combined treatment of blocking Tim-3 and PD-1 might be a better strategy for tumor immunotherapy, there are still more challenges and questions to be answered. First, there is a lack of valid biomarkers which can predict successful treatment with this combination currently. Recently studies showed that the even expression level of PD-L1 does not necessarily predict successful treatment (31, 98). Therefore, this combination should be approved on the basis of these biomarkers to limit ineffective treatments in the future. Next generation sequencing and nanostring analysis showing the results of total mutational burden or certain immune signatures present promising tests with the potential to discriminate between immune responsive or unresponsive patients, thus requiring further studies to confirm their utility as a predictive marker. Second, combinations will have to be patient tailored since they are likely to be more toxic than single agents and more expensive. Third, cells usually have functionally redundant pathways which could override and compensate for each other. Exhausted T cells upregulate several exhaustion markers (LAG-3,TIGIT and Tim-3 et al) and that targeting one of these will simply lead to an overexpression of another. The combination strategies should be carefully designed and should take into account T cell activation or exhaustion status. Notably, as compared to the two other immune checkpoints LAG-3 and TIGIT, Tim-3 is less validated and more robust clinical trials are still needed.

## Author Contributions

TT and ZL contributed to the conception of the study and wrote the manuscript. All authors contributed to the article and approved the submitted version.

## Funding

This study was supported by National natural Science Foundation of China (U1904139, 82070209 and U1804171), Department of Science & Technology of Henan province (182102310114).

## Conflict of Interest

The authors declare that the research was conducted in the absence of any commercial or financial relationships that could be construed as a potential conflict of interest.

## Publisher’s Note

All claims expressed in this article are solely those of the authors and do not necessarily represent those of their affiliated organizations, or those of the publisher, the editors and the reviewers. Any product that may be evaluated in this article, or claim that may be made by its manufacturer, is not guaranteed or endorsed by the publisher.
